# Severe Juxtahepatic Venous Injury: Survival after Prolonged Hepatic Vascular Isolation Without Shunting

**DOI:** 10.1155/1990/46171

**Published:** 1990

**Authors:** J. E. J. Krige, C. S. Worthley, J. Terblanche

**Affiliations:** 1 Department of Surgery and the Medical Research Council Liver Research Center University of Cape Town and Groote Schuur Hospital Cape Town South Africa; 2 Department of Surgery University of Cape Town Observatory 7925 South Africa

## Abstract

Survival following major juxtahepatic venous injury is rare in blunt liver trauma despite the use of intracaval shunting. Prolonged liver arterial inflow control, total hepatic venous isolation and lobectomy without shunting was used in a patient to repair a combined vena caval and hepatic venous injury after blunt liver injury. An extended period of normothermic hepatic ischemia was tolerated. Early recognition of retrohepatic venous injury and temporary liver packing to control bleeding and correct hypovolemia are essential before caval occlusion. Hepatic vascular isolation without shunting is an effective simple alternative technique allowing major venous repair in complex liver trauma.


*HPB Surgery*, 1990, Vol. 3, pp. 39–45
Reprints available directly from the publisher
Photocopying permitted by license only

© 1990 Harwood Academic Publishers GmbH
Printed in the United Kingdom

### SEVERE JUXTAHEPATIC VENOUS INJURY: SURVIVAL AFTER PROLONGED HEPATIC VASCULAR ISOLATION WITHOUT SHUNTING

J.E.J. KRIGE, C.S. WORTHLEY and J. TERBLANCHE
*Department of Surgery and the Medical Research Council Liver Research Center, University of Cape Town and Groote Schuur Hospital, Cape Town, South Africa.*

*(Received 11 January 1990)*

Survival following major juxtahepatic venous injury is rare in blunt liver trauma despite the use of intracaval shunting. Prolonged liver arterial inflow control, total hepatic venous isolation and lobectomy without shunting was used in a patient to repair a combined vena caval and hepatic venous injury after blunt liver injury. An extended period of normothermic hepatic ischemia was tolerated. Early recognition of retrohepatic venous injury and temporary liver packing to control bleeding and correct hypovolemia are essential before caval occlusion. Hepatic vascular isolation without shunting is an effective simple alternative technique allowing major venous repair in complex liver trauma.

KEY WORDS: Liver trauma, vena cava injury, hepatic vein injury, liver ischemia.

Uncontrolled bleeding due to major juxtahepatic venous injury is the leading intra-abdominal cause of death following blunt liver trauma[1]. Despite the widely recommended use of intracaval shunting as the optimal method for isolating the damaged retrohepatic vena cava and hepatic vein segments, mortality using this technique still exceeds 80% in experienced centres[2].

We report the successful use of prolonged liver arterial inflow occlusion and total hepatic venous isolation without shunting in the control and repair of a combined vena caval and hepatic vein injury following blunt liver trauma.

### CASE REPORT

A twenty-one year old newspaper vendor was admitted to hospital twenty minutes after being struck by a bus. He was shocked with a distended tender abdomen and had a positive peritoneal lavage. At laparotomy 1000 ml of free blood was present in the peritoneal cavity. Further exploration revealed a large stellate fracture with devitalization of the right lobe of the liver, disruption of the coronary ligament and extension of the laceration into the bare area and retrohepatic vena cava and hepatic veins. Active bleeding was controlled by temporary perihepatic packing and manual compression which allowed resuscitation. Bleeding persisted after

Correspondence to: J.E.J. Krige, Department of Surgery, University of Cape Town, Observatory 7925, South Africa.

removal of the packs, despite controlling inflow by clamping the hepatoduodenal ligament (the Pringle manoeuvre), suggesting a juxahepatic venous injury. The liver was repacked and exposure improved by performing a limited lateral thoracotomy via the right eighth intercostal space. Total duration of resuscitative perihepatic packing was 75 minutes. Seven units of blood were given during this period. Systolic blood pressure varied between 80 mm Hg and 140 mm Hg during the packing period. An experienced hepatic surgeon was summoned. Total hepatic venous isolation was achieved by clamping the infrahepatic vena cava above the renal veins and approaching the suprahepatic cava by incising the pericardium through the tendinous portion of the diaphragm. The suprahepatic cava was clamped within the pericardium and the infrahepatic cava controlled above the renal veins in conjunction with porta hepatis occlusion (the Pringle manoeuvre) (Figure 1). The devitalized right lobe was resected and the right hepatic artery was ligated within the liver tissue; arterial bleeding from smaller vessels at the resection margin was controlled by individual vessel suture ligation. The right hepatic vein had been avulsed from the inferior vena cava and the defect in the inferior vena cava was oversewn. A retrohepatic caval laceration extending into middle and left hepatic veins was repaired. Total intra-operative blood requirement including resuscitation was 18 units. Total duration of arterial inflow control was 2 hours and 50 minutes while total caval clamp time was 2 hours and 10 minutes. Mean systolic blood pressure was 50 mm Hg during the first 85 minutes and increased to 105 mm Hg during the remaining 45 minutes of the total caval clamp period.

A postoperative celiac arteriogram demonstrated a large right inferior phrenic artery supplying the residual left lobe and the left hepatic artery originating from the celiac axis. Hepatic venography 2 weeks following venous repair showed patent middle and left hepatic veins. The patient was discharged well 22 days after admission. The initially elevated liver enzymes returned to normal 10 weeks after the accident (Table 1). The patient remains well with patent middle and left hepatic veins on venography 2 years after the injury.

### DISCUSSION

Fifteen percent of patients with blunt liver trauma sustain hepatic venous injuries[1] and more than 80% die from uncontrollable hemorrhage, either before or during operation[3,4]. While minor juxtahepatic venous injuries can be repaired by direct suture using either digital compression[2] or partially occluding clamps, repair of major juxtahepatic venous injuries demands vascular control of both porta hepatis and retrohepatic venous segments[3]. Failure of portal triad occlusion to diminish major liver bleeding strongly suggests a juxtahepatic venous injury involving either the inferior vena cava or a major hepatic venous trunk[2,5]. Early recognition that a major juxtahepatic venous injury is present is essential since this necessitates a modification in the subsequent surgical approach[6].

The current operative techniques for vascular isolation of the injured liver use either internal atriocaval shunting or a multiple clamp non-shunting method. Since the introduction of the atriocaval shunt, initial reports of successful cases[3,4] and subsequently recent series[2,7] have added support for the use of the technique. The Houston group, who reported the first survivor with shunting[7], had an 81% mortality in 31 patients with major juxtahepatic venous injuries treated with atriocaval shunting[2]. No patient in their series who, in addition, required either

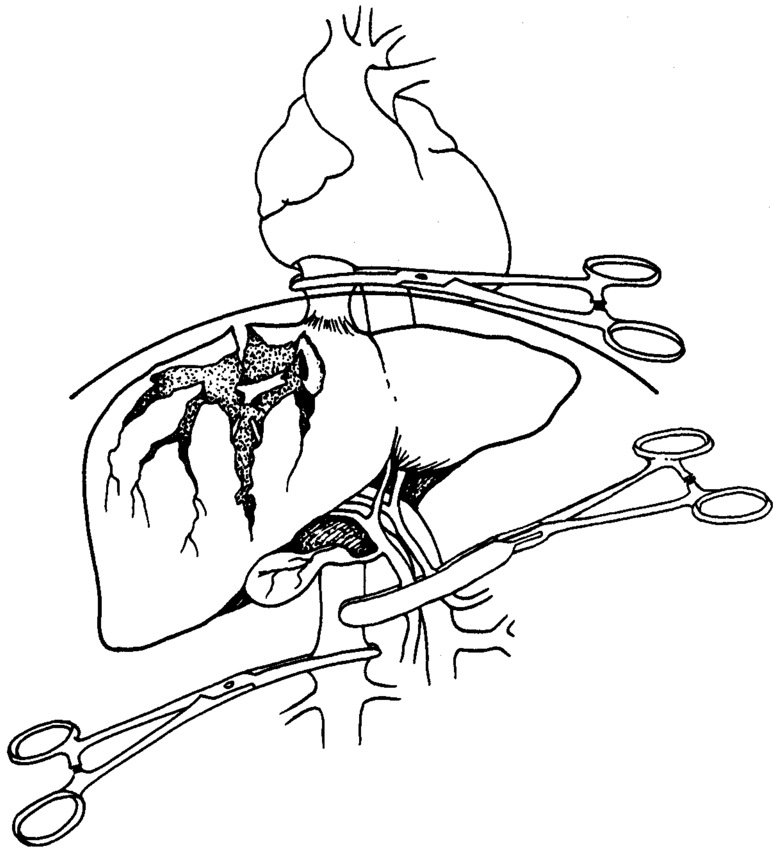

**Figure 1** Total hepatic vascular isolation with vena cava and porta hepatis control.

**Table 1** Liver Function Tests in the Patient after Prolonged Hepatic Ischemia.

| | *Normal Range* | *Day 2* | *Day 4* | *Day 6* | *Day 8* | *Day 15* | *Day 70* |
|---|---|---|---|---|---|---|---|
| LDH | (100–300u/L) | 1285 | 1248 | 1645 | 779 | 850 | 295 |
| ALT | (0–25u/L) | 562 | 453 | 303 | 196 | 68 | 22 |
| AST | (0–12u/L) | 920 | 298 | 129 | 66 | 59 | 10 |
| Total Bilirubin | (1–17mmol/L) | 13 | 48 | 33 | 42 | 28 | 15 |
| Alkaline Phosphate | (30–115u/L) | 65 | 111 | 92 | 112 | 105 | 72 |

resuscitative thoracotomy or liver resection, or in whom technical difficulties occurred with shunt insertion, survived[2]. The prohibitive mortality rates experienced with major juxtahepatic venous injuries treated with atriocaval shunting have provided the stimulus for simpler alternative techniques.

Total hepatic venous isolation without shunting using occluding clamps on both the suprahepatic and suprarenal vena cava in conjunction with a Pringle manoueuvre (Figure 1) was devised and used by Heany[8] and later by Huguet during complex elective liver resections[9]. This technique was subsequently applied in hepatic venous injury[3]. A critical caveat in the trauma situation is the prevention of cardiac arrhythmias which may follow complete caval occlusion. Adequate volume resuscitation during liver packing before clamping is essential to avoid this complication. If hypotension persists, venous bypass as used for hepatic transplantation is an option[10]. While additional aortic clamping has been recommended to avoid hypotension and peripheral pooling[3], this manoeuvre may compromise renal function and we strongly recommend that it not be used. Partial occlusion of the suprahepatic vena cava in children is well tolerated and facilitates repair[11]. A limited median sternotomy provides access to the suprahepatic cava through the tendinous central diaphragm and simplifies proximal caval control in adults[10].

Reluctance in the past to use prolonged inflow control by portal triad occlusion was based primarily on poor canine tolerance to hepatic ischemia[12]. Recent clinical data has extended the traditional concept of limited hepatic ischemic tolerance[13]. The use of normothermic total hepatic vascular occlusion for as long as 65 min during extensive elective hepatic resection is well tolerated[9, 12], while hepatic ischemia lasting 90 min following inadvertant portal triad division in a patient had no untoward effects[13]. Support for the extension of the safe period in the trauma context with successful occlusion of the portal triad for more than 1 hr in the management of hemorrhage from complex liver injuries is reported[6] The anomalous blood supply to the residual lobe in our patient may have provided a beneficial effect allowing more prolonged liver tolerance to warm ischemia than we would normally advocate.

The crucial factors in the operative management of juxtahepatic venous injuries are early identification and urgent control of bleeding[11]. Major posterolateral stellate fractures with disruption of the coronary ligament and extension into the bare area with profuse bleeding suggest caval or hepatic venous injury[11]. Adequate resuscitation after packing is fundamental[14]. Failure to control bleeding after inflow occlusion confirms a retrohepatic venous injury. Total vascular isolation of the liver without shunting provides an effective alternative technique for juxtahepatic venous repair.

### *Acknowledgements*

Financial assistance is acknowledged from the South African Medical Research Council and the University of Cape Town Staff Research Fund.

### *References*

1. Schrock, T. and Blaisdell, F.W. (1968) Management of blunt trauma to the liver and hepatic veins. *Arch. Surg.* **96**, 698–704.
2. Burch, J.M., Feliciano, D.V. and Mattox, K.L. (1988) The atriocaval shunt: facts and fiction. *Ann.Surg.* **207**, 555–568.
3. Yellin, A.E., Chaffee, C.B. and Donovan, A.J. (1971) Vascular isolation in treatment of juxtahepatic venous injuries. *Arch. Surg.* **102**, 566–573.
4. Bricker, D.L., Morton, J.R., Okies, J.E. *et al.* (1971) Surgical management of injuries to the vena cava: changing patterns of injury and newer technique of repair. *J. Trauma* **11**, 725–735.
5. Kudsk, K.A., Sheldon, G.F. and Lim, R.C. (1982) Atrial-caval shunting after trauma. *J. Trauma* **22**, 81–85.

6. Pachter, H.L., Spencer, F.C., Hofstetter, S.R., Liang, H.C. and Coppa, G.F. (1986) The management of juxtahepatic venous injuries without an atriocaval shunt: preliminary clinical observations. *Surgery* **99**, 569–575.
7. Bricker, D.L. and Wukasch, D.C. (1970) Successful management of an injury to the suprarenal inferior vena cava. *Surg.Clin.North.Am.* **50**, 999–1002.
8. Heany, J.P., Stanton, W.K., Halbert, D.S. *et al.* (1966) An improved technic for vascular isolation of the liver: experimental study and case reports. *Ann.Surg.* **163**, 237–241.
9. Huguet, C., Gallot, D. and Offenstadt, G. (1976) Normothermic complete hepatic vascular exclusion for extensive resection of the liver. *N.Engl J Med* **294**, 51–52.
10. Shaw, B.W., Martin, D.J., Marquez, J.M. *et al.* (1984) Venous bypass in clinical liver transplantation. *Ann. Surg.* **200**, 524--534.
11. Coln, D., Crighton, J. and Schorn, L. (1980) Successful management of hepatic vein injury from blunt trauma in children. *Am. J. Surg.* **140**, 858–864.
12. Huguet, C., Nordlinger, B., Bloch, P. and Conard, J. (1978) Tolerance of the human liver to prolonged normothermic ischaemia. *Arch.Surg.* **113**, 1448–1451.
13. Kahn, D., Hickman, R., Dent, D.M. and Terblanche, J. (1986) For how long can the liver tolerate ischaemia? *Eur.Surg.Res.* **18**, 277–282.
14. Terblanche, J. and Krige, J.E.J. (1990) Injuries to the liver and bile ducts. In: Acute Abdominal Emergencies, edited by R. Williamson and M. J. Cooper, London: Churchill Livingstone (In press)

(*Accepted by S. Bengmark on 11 January 1990*)

### INVITED COMMENTARY

Major juxtahepatic venous injury with uncontrollable bleeding is a most serious situation in which several modalities of treatment have been employed.

a. Effective packing and tamponade.
b. Intracaval shunting and repair.
c. Transplantation.

The present paper describes an approach, including vascular isolation and prolonged warm ischemia during which resection of non-vital tissue and venous repair could be accomplished. This case report is of importance since treatment of blunt liver trauma requires an approach that is determined by institutional prerequisites, such as availability of a trauma service, bypass availability, transplant set up and specialized trauma as well as hepatobiliary surgical expertise.

Major trauma centers are presenting series of successful treatment of juxta venous injury with the atriocaval shunt procedure. It is however, not clear what particular circumstances prompt their choices of employing the internal bypass, which is technically no different from a total vascular exlusion procedure, but has the advantage of providing venous blood return during the phase of hypertension and possible cardiac failure. This can be accomplished as well by an external bypass, as used by the transplant groups. Since the transplant surgeons are not necessarily involved in trauma cases of that severity, the use of internal bypass and repair, or occasionally transplantation, has only been reported in selected cases (lit).

The approach of this Capetown Group is essentially not new since vascular exclusion, Pringle maneuver or selected hepatic artery ligation and resection is routine practice in elective hepatic surgery. To apply this expertise to a rare case is, however, exemplary and should serve as a model for closer effective intersurgical cooperation between trauma surgeons and hepatobiliary surgeons. This paves the way to the coordinated and timely application of advanced technology in an escalation of complications, such as the presented case with liver injury, followed by rapid diagnosis and resuscitation, laparotomy and tamponade, resuscitation again, vascular exclusion and resection and finally, control of hemorrhage.

Unfortunately, the majority of patients don't arrive at that stage simply because their bleeding could either be controlled by packing, or shock events and blood loss have lead to cardiac arrest and secondary organ failures. Thus, there is no defined line between continuation of the surgical attempt at repair and institution of a bypass.

In the situation described however, there was obvious time to continue resuscitation and a plan for definite surgical repair. Given the severity of the injury this is uncommon and the argument of trauma specialists is that, particularly during the hypotensive phases, the bypass is crucial and should be employed as early as possible, even if theoretically, it could be prevented. Whatever procedure is employed, it is important that a management plan should be in place and executed promptly.

All previous experience indicates that the authors took their chance by extension of the warm ischemia time beyond the usual accepted length of about one hour without cold ischemic protection of the tissue. To conclude from this one case that this procedure is applicable to different situations is premature. Rather, the opposite should be concluded since there was no obvious clue described, according to which a choice of one or the other methods could be made. At the time of their decision, the authors had no means to assess the reversibility or severity of the ischemic damage and to simply take more than two hours to repair the injury without a bypass, seems to be rather desperate than based on knowledge of ischemic tolerance of the liver. The argument could be made that, despite technical success in controlling the hemorrhage, liver failure would be the inevitable outcome and, therefore, the situation would have called for a transplant. It is somewhat surprising that the patient was not in renal failure or pulmonary failure, which indicates that the hemodynamic and ventilatory situation was never critical or out of control. The functional preservation of the kidney provides an estimate of the pre-surgical shock episode and the degree of intraoperative hypotension. Another factor assisted the authors to succeed with such a long ischemic time: The presence of an aberrant left artery most likely accounts for maintaining a residual blood flow through some parts of the left lobe, thus, preventing total necrosis.

Of considerable interest is the monitoring of liver function following the ischemic event. While enzyme release within the first 24 hours provides little information, due to a 'washout effect' following massive transfusion, the subsequent rise of enzymes in serum bilirubin provides an estimate as to the degree of damage. The serum bilirubin of 48 U/L (about 2.5mg/dl), indicates recoverable jaundice and no evidence of sepsis. Thus, recuperation could almost certainly be anticipated. The lesson from the transplant experience indicates that enzyme release of more than 10,000 U/L and/or a subsequent bilirubin rise to more than 50mg/dl would be consistent with liver failure. It should be stressed that these parameters are rough estimates - though valid data - suggest liver failure and prompt a search for a transplant organ. Further specific tests such as MEGX or Indocine Green clearance should be advocated to monitor liver function following major ischemic episodes.

In summary, hepatic venous repair can be successfully performed provided the management plan is in place, using either one of the available technologies at the earliest time of necessity. Involvement of hepatobiliary and trauma surgical specialists is advocated.

Professor Christoph E. Broelsch
Department of Surgery
The University of Chicago
Box 259
5841 South Maryland Avenue
CHICAGO, Il 60637
USA

### *References*

Busuttil, R.W., Kitahama, A., Cerise, E., McFadden, M., Lo, R., Longmire, W.P., Jr. (1980) Management of blunt and penetrating injuries to the porta hepatis. *Ann.Surg.*, **191**, 641–648.

Cheatham, J.E., Jr., Smith, E.I., Tunell, W.P., Elkins, R.C. (1980) Nonoperative management of subcapsular hematomas of the liver. *Am.J.Surg.*, **140**, 852–857.

Coln, D., Crighton, J., Schorn, L. (1980) Successful management of hepatic vein injury from blunt trauma in children. *Am.J.Surg.*, **140**, 858–864.

Elerding, S.C., Aragon, G.E., Moore, E.E. (1979) Fatal hepatic hemorrhage after trauma. *Am. J. Surg.*, **138**, 883–888.

Fabian, T.C., Mangiante, E.C., White, T.J., Patterson, C.R., Boldreghini, S., Britt, L.G.: (1986) A prospective study of 91 patients undergoing both computed tomography and peritoneal lavage following blunt abdominal trauma. *J. Trauma*, **26**, 602–608.

Fabian, T.C., Stone, H.H. (1980) Arrest of severe liver hemorrhage by an omental pack. *Southern Med. J.*, **73**, 1487–1490.

Feliciano, D.V., Mattox, K.L., Burch, J.M., Bitondo, C.G., (1986) Packing for control of hepatic hemorrhage. *J. Trauma*, **26**, 738–743.

Feliciano, D.V., Mattox, K.L., Jordan, G.L., Jr., Burch, J.M., Bitondo, C.G., Cruse, P.A. (1986) Management of 1000 consecutive cases of hepatic trauma (1979–1984). *Ann. Surg.*, **204**, 438–445.

Franklin, R.H., Bloom, W.F., Schoffstall, R.O. (1980) Angiographic embolization as the definitive treatment of post-traumatic hemobilia. *J. Trauma*, **20**, 702–705.

Geis, W.P., Schulz, K.A., Giacchino, J.L., Freeark, R.J. (1981) The fate of unruptured intrahepatic hematomas. *Surgery*, **90**, 689–697.

Ivatury, R.R., Nallathambi, M., Gunduz, Y., Constable, R., Rohman, M., Stahl, W.M. (1986) Liver packing for uncontrolled hemorrhage: A reappraisal. *J. Trauma*, **26**, 744–753.

Kudsk, K.A., Sheldon, G.F., Lim, R.C., Jr. (1982) Atrial-caval shunting (ACS) after trauma. *J. Trauma*, **22**, 81–85.

Mays, E.T. (1976) The hazards of suturing certain wounds of the liver. *Surg. Gynecol. Obstet.*, **143**, 201–203.

Oldham, K.T., Guice, K.S., Ryckman, F., Kaufman, R.A., Martin, L.W., Noseworthy, J. (1986) Blunt liver injury in childhood: Evolution of therapy and current perspective. *Surgery*, **100**, 542–549.

Pachter, H.L., Spencer, F.C., Hofstetter, S.R., Coppa, G.F. (1983) Experience with the finger fracture technique to achieve intra-hepatic hemostasis in 75 patients with severe injuries of the liver. *Ann. Surg.*, **197**, 771–778.

Pachter, H.L., Spencer, F.C., Hofstetter, S.R., Liang, H.C., Coppa, G.F. (1986) The management of juxtahepatic venous injuries without an atriocaval shunt: Preliminary clinical observations. *Surgery*, **90**, 569–575.

Peitzman, A.B., Makaroun, M.S., Slasky, B.S., Ritter, P. (1986) Prospective study of computed tomography in initial management of blunt abdominal trauma. *J. Trauma*, **26**, 585–592.

Sheldon, G.F., Lim, R.C., Yee, E.S., Peterson, S.R. (1985) Management of injuries to the porta hepatis. *Ann.Surg.*, **202**, 539–545.

Schmidt, B., Bhatt, G.M., Abo, M.N. (1980) Management of post traumatic vascular malformations of the liver by catheter embolization. *Am.J.Surg.*, **140**, 332–335.

